# The costs of hepatitis A infections in South Korea

**DOI:** 10.4178/epih/e2014011

**Published:** 2014-08-18

**Authors:** Kyohyun Kim, Baek-Geun Jeong, Moran Ki, Mira Park, Jin Kyung Park, Bo Youl Choi, Weon-Seob Yoo

**Affiliations:** 1 Research Institute, Health Insurance Review & Assessment Service, Seoul, Korea; 2 Department of Preventive Medicine, Gyeongsang National University School of Medicine, Jinju, Korea; 3 Department of Cancer Control and Policy, Graduated School of Cancer Science and Policy, National Cancer Center, Goyang, Korea; 4 Department of Preventive Medicine, Eulji University School of Medicine, Daejeon, Korea; 5 International Vaccine Institute, Seoul, Korea; 6 Department of Preventive Medicine, Hanyang University College of Medicine, Seoul, Korea; 7 Regional Cardiocerebrovascular Center, Chungnam National University Hospital, Daejeon, Korea

**Keywords:** Cost of illness, Hepatitis A, Infection, Republic of Korea

## Abstract

**OBJECTIVES:**

The incidence of hepatitis A infections among young adults has recently increased in South Korea. Although universal vaccination has often been suggested to mitigate the problem, its rationale has not been well-understood. Estimating the societal costs of hepatitis A infections might support the development of intervention strategies.

**METHODS:**

We classified hepatitis A infections into eight clinical pathways and estimated the number of occurrences and cost per case for each clinical pathway using claim data from National Health Insurance and several national surveys as well as assumptions based on previous studies. To determine the total costs of a hepatitis A infection, both direct and indirect costs were estimated. Indirect costs were estimated using the human-capital approach. All costs are adjusted to the year 2008.

**RESULTS:**

There were 30,240 identified cases of hepatitis A infections in 2008 for a total cost of 80,873 million won (2.7 million won per case). Direct and indirect costs constituted 56.2% and 43.8% of the total costs, respectively. People aged 20-39 accounted for 71.3% of total cases and 74.6% of total costs. Medical costs per capita were the lowest in the 0-4 age group and highest in the 20-29 age group.

**CONCLUSIONS:**

This study could provide evidence for development of cost-effective interventions to control hepatitis A infections. But the true costs including uncaptured and intangible costs of hepatitis A infections might be higher than our results indicate.

## INTRODUCTION

Hepatitis A is an acute infectious disease that exhibits symptoms including jaundice, fever, and loss of appetite. In general, the disease spreads through human-to-human, foodborne, and waterborne transmission [1]. Over 1.4 million cases of hepatitis A occur worldwide each year. Thus, the economic burdens associated with the disease are estimated to be between $1.5 to 3.0 billion per year [2].

Since the 1990s, many countries have faced decreasing natural infection occurrences during childhood because of the increase in socioeconomic and hygiene levels. However, such reduced natural infections also reduced the rate of the hepatitis A antibodies in the overall population, resulting in an epidemiological transition of increasing hepatitis A susceptibility in adult populations [1,3,4]. Contrary to pediatric hepatitis A, adult hepatitis A is generally presented as a symptomatic infection and can cause death. Therefore, the increase in the number of individuals susceptible to hepatitis A in the adult population is a looming global public health issue [5].

South Korea has also experienced an epidemiological transition of hepatitis A in the past 10 years. Echoing the increasing concern, a significant proportion of the adult population lacks hepatitis A antibodies [6-9]. According to the Korea Centers for Disease Control and Prevention, there were approximately 8,000 hepatitis A incidences in 2008, representing a 20-fold increase compared to 2004. Approximately 80% of cases in 2008 occurred in the adult population aged between 20 and 39 years. Based on this data, it is clear that responsive hepatitis A intervention strategies must be urgently developed [10].

Although universal vaccination has been suggested as a method for managing hepatitis A in South Korea, evidence in support of a vaccination strategy are currently lacking [11,12].

The present study was conducted to present the socioeconomic costs of hepatitis A in South Korea in 2008 to capture the attention of health authorities and the general public regarding the disease by provding necessary evidences for developing a cost-effective intervention strategy to manage hepatitis A.

## MATERIALS AND METHODS

### Research design and data collection

This study used an incidence-based approach to estimate the economic burden of hepatitis A infections in South Korea in 2008. The incidence-based approach is a method that computes the cost of the disease over the lifecycle of a single incident from the start of the incident to complete recovery or death. This method can be used to estimate the benefit realized when a single occurrence of the disease is prevented [13,14]. The disease burden of hepatitis A infections was estimated using the following three steps.

1. We first classified hepatitis A infections into eight clinical pathways and calculated the incidence rate of each clinical pathway by age group. We multiplied this incidence rate by the number of hepatitis A incidences by age group to compute the total number of incidences for each age group and clinical pathway.
2. Then, we segmented the total cost of a single hepatitis A case into direct and indirect costs and computed the cost of a single case for the respective age group and clinical pathway.
3. We multiplied the number of incidences in the respective age group and clinical pathway (outcome of step 1) with the cost of hepatitis A per person (outcome of step 2) to determine the total social cost of hepatitis A.

#### National Health Insurance claims data

In order to estimate the number of hepatitis A incidences and their associated costs in a single year (2008) in South Korea, we used National Health Insurance (NHI) claims data. National Health Insurance Corporation stores the demographic information, diagnostic codes, dates of consultation and treatment, treatment fees, and death records of NHI enrollees through computerized management. If these data are used, it is possible to conduct studies of the cost of a particular disease on a national level. Costs of disease studies regarding asthma, injuries, strokes, and hepatitis B using this data set have already been reported [15-18].

The present study used NHI claims data and identified approximately 80,000 claims of hepatitis A infections (International Classification of Diseases version 10 codes B15, B15.0, B15.9) using primary or secondary diagnostic codes. The claim data were then converted to 30,240 cases of person-based data, which allows measurement of the total treatment period and cost for each individual. Because hepatitis A is a disease that produces immunity after a single infection, each unit of person-based data can be regarded as a single case of hepatitis A.

The data used for analysis were provided by the Health Insurance Review and Assessment Service, which processed the data to encrypt any personal identifiers. All matters associated with the conduct of the present study were approved by the institutional review board (No. 09-09) of Eulji University Hospital.

### Hepatitis A incidence rate and number of incidences with respect to age group and clinical pathway

**Figure 1.**
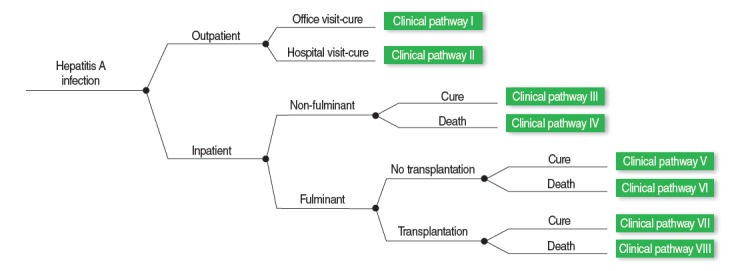
Eight clinical pathways model of hepatitis A infections.

The present study is based on a previous study in that it categorized hepatitis A infections in eight mutually exclusive clinical pathways depending on the status of hospitalization, fulminant hepatitis incidence, liver transplantation, and death (Figure 1). Clinical pathways I and II are hepatitis A infections that were cured only through outpatient treatment. Pathway I indicates visits to a clinic, whereas pathway II indicates visits to a hospital. Clinical pathways III and IV are cases in which patients had survived and died, respectively, after being hospitalized because of non-fulminant hepatitis. Clinical pathways V and VI are cases in which patients had survived and died, respectively, after being hospitalized because of fulminant hepatitis but were not able to receive liver transplantations for various reasons. Clinical pathways VII and VIII are cases in which patients had survived and died, respectively, after being hospitalized because of fulminant hepatitis and receiving liver transplantations.

The incidence rate of the respective clinical pathways can be calculated by multiplying the probabilities at points where the respective clinical pathways bifurcate (Figure 1). For example, if the probabilities of hospitalization because of hepatitis A, fulminant hepatitis incidence in an inpatient, liver transplantation for inpatients with fulminant hepatitis, and survival for patients that had received the liver transplantation are known, the probability at each step can be multiplied to compute the incidence rate of clinical pathway VIII in a hepatitis A patient. NHI claims data and precedent study results were used to compute the probabilities at each bifurcation point for the respective age groups. In the case that probabilities for each age group could not be computed, we assumed that the probabilities at a given bifurcation point were the same for all age groups.

The probability of an outpatient visit (clinic, hospital) and the probability of hospitalization with respect to each age group were computed using NHI claim data. The incidence rate of fulminant hepatitis for inpatients was set to 1.1% as reported by Bauch et al. [19], because no domestic data were available. The liver transplantation rate of fulminant hepatitis patients (37.1%), case fatality rate of liver transplantation patients (23.1%), and case fatality rate of patients that did not receive liver transplantations (9.1%) were reported from domestic study results [20]. The probability of death for a non-fulminant hepatitis patient was computed by subtracting the probability of death for a fulminant hepatitis patient from the probability of death for a hepatitis patient overall [20,21].

The probabilities calculated in this manner were multiplied by the number of hepatitis A incidences with respect to age groups according to the claims data to compute the number of hepatitis A incidences with respect to age group and clinical pathway. Meanwhile, we computed the incidence rate of hepatitis A with respect to age group using the population data from the Korea National Statistical Office (KOSIS).

### Cost of hepatitis A estimation

The cost of hepatitis A was segmented into direct and indirect costs with respect to age groups and clinical pathways. Direct costs include medical, supplemental care, and transportation costs. Indirect costs included productive losses due to treatment or premature death. All costs were adjusted to 2008 using the June 2008 exchange rate (1 USD=1,000 Korean won) and displayed in USD.

#### Direct cost

Medical costs were categorized as costs paid by insurance, by patients under copayment, and by patients for services not covered. Costs paid by insurance and by patients under copayment were labeled costs for covered services and computed using claims data and precedent studies. Hepatitis A costs paid by patient for non-covered services were computed using results from “the state of out-of-pocket medical cost burdens of a patient covered by the NHI in 2007” to apply the ratio between “cost paid by the patient for covered services” to “cost paid by the patient for non-covered services” [22]. Meanwhile, NHI claims data used in the present study did not include costs of outpatient medication. Thus, we indirectly computed the total cost of outpatient medication using the number of prescription days, number of visit days, and cost of medication per day in outpatients in the 2007 NHI yearly statistics[23].

Medical costs for clinical pathways I and II were computed by selecting claims that mapped hepatitis A cases without records of inpatient treatment. These medical costs were computed for each age group. The medical costs of clinical pathways III and IV were computed by selecting claims with a hospitalization record, whose medical cost resulting from the disease were below the top 1.1%, and who posted a treatment result of either “survival” or “death.” The 1.1% threshold used in the calculation is based on the results of a preceding study that reported that the proportion of fulminant hepatitis cases in all hepatitis cases is 1.1% and the assumption that fulminant hepatitis has a higher medical cost than non-fulminant hepatitis does [19].

Medical costs of clinical pathways V and VI were computed by multiplying the number of inpatient days extracted from a domestic precedent study and the daily medical cost of hospitalization computed in clinical pathway III [20]. Thus, the medical costs of clinical pathways V and VI were $7,157 and $9,169, respectively.

Medical costs of clinical pathway VII were calculated based on a Korean precedent study associated with liver transplantation costs. Yang et al. [18] reported liver transplantation costs of a hepatitis B patient, and Ha [24] compared liver transplantation costs between hepatitis B patients and other patients. Using this information and accounting for the rise in medical fee schedule, liver transplantation costs for a hepatitis A patient in 2008 was calculated. Using this method, first-year liver transplantation inpatient costs, first-year outpatient costs, and second-year inpatient costs were $72,850, $4,203, and $9,279, respectively. Thus, the total cost of clinical pathway VII was $86,332.

The medical costs of clinical pathway VIII were calculated based on the number of hospitalization days of the transplantation patient and on the medical costs of clinical pathway VII. We first extracted the medical cost per inpatient day using the number of inpatient days spent by a hepatitis A patient that survived after liver transplantation as well as the inpatient costs of clinical pathway VII. Then, we multiplied the computed medical cost per inpatient day by the number of inpatient days spent by a hepatitis A patient that did not survive after liver transplantation to obtain the inpatient cost for a hepatitis A patient that did not survive the liver transplant [20]. Thus, the computed medical costs of clinical pathway VIII were $40,246.

The non-medical direct costs of hepatitis A patients included transportation costs and supplemental care costs incurred from providing extra care to inpatients. Transportation costs were calculated by obtaining the sum of inpatient transportation costs and the number of outpatient visit days multiplied by the transportation cost per outpatient visit day, which was calculated in the following manner: First, we used the national database “2005 Korea National Health and Nutrition Examination Survey” to calculate the round-trip cost of an outpatient clinic visit, an outpatient hospital visit, and inpatient transportation. Then, we accounted for the consumer price index (CPI) to calculate transportation costs at the 2008 price level. The outpatient clinic visit and outpatient hospital visit transportation costs were $5.0 and $10.1, respectively. The transportation cost for a hospitalization case was $23.5. The calculation of outpatient visit days is explained in the indirect cost calculation method below.

Supplemental care costs are direct costs incurred from providing extra care beyond the scope of care covered by standard medical costs. By accounting for the standard domestic situation regarding supplemental care, we assumed that supplemental care costs are incurred daily in a fixed amount for the duration of inpatient stay [25,26]. We set daily supplemental care costs to $40 based on the market price in 2008.

#### Indirect costs

With respect to the indirect costs, we used the human capital approach to estimate the cost of productive loss due to hepatitis A. The cost of productive loss for the duration of the treatment period (because of morbidity) was calculated by multiplying the number of non-productive days because of hepatitis A by the average daily wage. The number of non-productive days was calculated by adding the number of inpatient days and one-third of the number of outpatient visit days [16,17,25].

The number of inpatient and outpatient visit days for clinical pathways I through IV were calculated using NHI claims data. The number of inpatient days for clinical pathways V and VI were set to 18.5 and 23.7 days, respectively, based on previous studies. Outpatient visit days for clinical pathways V and VI were computed by multiplying the number of inpatient days by the number of outpatient visit days per inpatient day given by clinical pathway III. As a result, the outpatient visit days of clinical pathways V and VI were 3.2 and 4.1 days, respectively [20]. The number of inpatient days for clinical pathways VII and VIII were 42.9 and 23.7 days, respectively, based on the same aforementioned study. The outpatient visit days of clinical pathway VII were calculated under the assumptions of two outpatient visit days per month for the first 6 months after liver transplantation, 1 day per month for the next 12 months, and no outpatient visits thereafter. Thus, the number of outpatient visit days for clinical pathway VII was calculated as 24.6 days. We assumed that there were no outpatient visit days for clinical pathway VIII. The average daily wage adjusted to 2008 was computed by reformulating the total monthly wage by age group in 2006 to the average wage. Then, we accounted for the rising CPI and employment rate by age group to compute the final average daily wage [27]. Individuals between the ages 15 and 69 were assumed to be engaged in economically productive activities. Average daily wages of a 15-year old individual was substituted with the average daily wages of a 16-year old, because wage statistics do not evaluate wages earned by 15-year olds. Furthermore, we used the average daily wage of a 60-year old individual to account for the average daily wage of individuals between the age of 61 and 69. Productive losses incurred from outpatient visits by individuals under the age of 14 or over the age of 70 were calculated by assuming that the guardian of the aforementioned age groups was a 40-year old female. Thus, we estimated productive losses by applying the average daily wage and the employment rate of care giver’s age group.

The productive losses incurred from premature death (because of mortality) was calculated by estimating the future expected income of the deceased patient under the assumption that he or she would have survived to work until the age of 69. We considered the average annual wages, employment rate, and natural death rate by age group in 2008 and applied a 5% annual discount rate to calculate the future expected income in 2008 dollars [27]. The average annual wages by age group in 2008 were calculated by multiplying the 2006 wages provided by KOSIS by the increasing annual CPI. The natural death rate was also acquired from KOSIS. Using this methodology, the cost of productive losses for the death of a 30-year old in 2008 was $378,000.

$$\text{Costs of productive loss due to mortality}=\sum_{i=0}\sum_{\tau =0}\frac{N_{i}\times w_{i+\tau }\times e_{i+\tau }\times d_{i+\tau }}{{\left(1+r\right)}^{\tau }}\;\text{i: age of mortality case}\;\text{τ: years after death}\;\text{ω: annual wage rate}\;\text{e: employment rate}\;\text{d: natural mortality rate}\;\text{r: discount rate (0.05)}$$

### Sensitivity analysis

In order to perform a sensitivity analysis, we considered the fatality of non-fulminant hepatitis A and the probability of receiving a liver transplantation surgery as a fulminant hepatitis A patient. When the aforementioned metrics changed to 50% or 150% of their default values, we presented the change in the magnitude of the hepatitis A cost burden in the results.

## RESULTS

### The number of hepatitis A incidences

The number of hepatitis A incidences in South Korea in 2008 identified by NHI claims data was 30,240 cases. The incidence rate was 62.2 cases per 100,000 individuals (Table 1). There were 10,643 and 10,902 cases for age groups 20-29 and 30-39, respectively, accounting for 71.3% of the total number of incidences. The incidence rates for age groups for age groups 20-29 and 30-39 were the highest at 148.2 and 131.6 cases per 100,000 individuals, respectively. Regarding the number of incidences by clinical pathway, clinical pathway III (i.e., non-fulminant hepatitis cured through inpatient treatment) was the highest and most common: 18,075 cases accounting for 59.8% of the total number of incidences. It was estimated that 202 hepatitis A cases had fulminant hepatitis, 75 of which resulted in a liver transplantation surgery.

With respect to the probability of hepatitis A by age group and clinical pathway, the probability of an incidence with clinical pathways III-VIII requiring hospitalization was the highest in the 20-29 age group at 68.8%, followed by the 5-9 and 60-69 age group with probabilities 30.3% and 25.0%, respectively. The probability of a hepatitis incidence for clinical pathways IV, VI, and VIII, indicating death, was lowest in the 0-4 age group at 0.1% and highest in the 80-89 age group at 3.9%.

### Cost per hepatitis A case

The total cost of a single case of hepatitis A, including direct and indirect costs, was $2,674. With respect to age group, the total cost per case was the lowest in the 0-4 age group at $934. By contrast, the total cost per case was highest in the 40-49 age group at $3,046 (Table 2). Regarding the clinical pathways, the lowest total costs per case were found in clinical pathways I and II at $162 and $259, respectively (because of their lack of hospitalization). Clinical pathway VII (i.e., a complete recovery after liver transplantation) imposed a cost of $91,001 per case. The costs per case were highest in clinical pathways IV, VI, and VIII (indicating patient death) at $200,000. For the most common hepatitis A incidence type (clinical pathway III for the 20-29 age group), the total cost per case was $2,489.

Within the total average cost of $2,674 for a hepatitis A case, direct costs were $1,502, where medical costs amounted to $1,206. Given the same clinical pathway, the total cost of a single case of hepatitis A still differed depending on the age group. In the case of clinical pathway III, the medical cost per case was approximately 1.5 times higher in the age group 20-39 compared to that of the age group 0-4.

### The total cost of hepatitis A

In the year 2008, the total cost incurred because of hepatitis A incidences was estimated to be approximately $80,873,000 (Table 3). Given the total cost of hepatitis, direct and indirect costs accounted for 56.2% and 43.8% of the total cost, respectively. Overall, 80.3% of the direct costs were medical costs, whereas the remaining 19.3% consisted of supplemental care costs and transportation costs. In total, 59.3% of the medical costs were paid by health insurance, whereas 40.7% was paid by the patients. One-third of the indirect costs were productive losses incurred from using the medical services, and the remaining two-thirds of the indirect costs were productive losses incurred from early death.

Clinical pathway III constituted 58.3% of total costs incurred by hepatitis A, capturing the largest cost among the eight clinical pathways. The total number of outpatient visits because of hepatitis A was 64,031, and the total number of inpatient days was 198,715 (i.e., 544 years).

The total cost incurred in 2008 because of hepatitis A distributed by age group is presented in Table 4. In the total cost distribution by age group, the 30-39 age group incurred the highest cost at 39.4% of all costs. Costs incurred by people aged 20-49 were 87.4% of the total cost.

### Sensitivity analysis results

Sensitivity analysis results are presented in Table 5. When both the fatality of a hepatitis A infection and the probability of transplantation increased by 50% (sensitivity analysis 6), the total cost of hepatitis A infections was $97,262,000, 20.3% higher than the baseline analysis. When the probability of fatality from a hepatitis A infection and that of transplantation decreased to 50% of the baseline (sensitivity analysis 5), the total cost of hepatitis A infections was calculated at $64,484,000, 20.3% lower than the baseline analysis. Change in the fatality level of non-fulminant hepatitis A had a greater influence on the total cost than the change in transplantation probability did.

## DISCUSSION

The present study estimated the economic costs of hepatitis A in South Korea in 2008 by an incidence-based approach using NHI claims data. The number of hepatitis A incidences in 2008 was 30,240. From a socioeconomic perspective, the total cost of the disease was $80,873,000. Medical and non-medical direct costs respectively represented 45.1% and 11.1% of total costs. Indirect costs were 43.8% of total costs. The 20-39 age group constituted 71.3% of the total number of incidences and 74.6% of the socioeconomic costs. The cost per case of hepatitis A was calculated to be $2,674, which is lower than that of other diseases (e.g., cancer, stroke, asthma, or injury) reported in South Korea [16,17,26,28]. Compared to other digestive diseases, the cost of hepatitis A is higher than that of rotavirus (another digestive infectious disease) and is lower than that of irritable bowel syndrome [29,30]. The total societal cost of the rotavirus in South Korea in 2005 including indirect costs was reported to be approximately $13,300,000. Accounting for the rise in CPI, the total societal cost of rotavirus in 2008 was $14,600,000. Comparatively, the total social cost of hepatitis A was approximately 5.5 times higher. Furthermore, the total societal cost of irritable bowel syndrome in 2008 was $585,400,000, 7.2 times higher than that of hepatitis A. The portions of the total costs of rotavirus and irritable bowel syndrome captured by indirect costs were 5% and 25%, respectively, markedly lower than those of hepatitis A (44%).

The 30,240 cases of hepatitis A incidences in 2008 found through the present study were confirmed solely through primary and secondary diagnostic codes in the claims data of the NHI. The claims data can influence the number of incidences and disease costs depending on how medical institutions report cases of hepatitis A to National Health Insurance Corporation. According to our unpublished data, there was no significant difference between the number of hepatitis A incidences identified through medical records from 2002 to 2008 in 11 general-hospital-level medical institutions (3,081 cases) and those identified from the NHI claims data (2,928 cases) for the same period. Thus, we determined that it was feasible to use the number of hepatitis A incidences identified through the NHI claims data as the number of hepatitis A incidences [31]. However, because we could not confirm how medical institutions other than general hospitals recorded hepatitis A cases, additional studies should be conducted in the future to provide a more accurate estimate of the number of hepatitis A incidences.

The total societal cost of hepatitis A in South Korea in 2008 was estimated to be 0.008% of the gross domestic product (GDP) of South Korea and 0.12% of national health expenditures. In a US study published in 2000, the total societal cost of hepatitis A in the US was estimated to be $48,880,000, 0.006% of the US GDP and 0.044% of national health expenditures that year. The total societal cost of hepatitis A in the US was also relatively lower than that of South Korea [32]. Such statistics can partially be explained by the fact that the number of Korean hepatitis A incidences reported in the present study was approximately two times greater than that of the number reported in the aforementioned US study. It was also reported that the portion of the total costs of hepatitis A captured by indirect costs was 44% in South Korea, whereas it was 74% in the US, relatively conservative estimate. A lack of data on countries other than the US meant that we could not directly compare the total societal cost of hepatitis A in these countries with such costs in South Korea as calculated in the present study.

As hepatitis A infections occurring in the age group 20-39 constitutes over 70% of the total number of incidences and societal costs, an intervention strategy to lower the total societal cost of hepatitis A should involve the management of hepatitis A in young adults. Although prioritizing an effective hepatitis A vaccination of young adults initially seems like an ideal solution, a national pediatric vaccination effort has been reported to inhibit transmission by asymptomatic patients and induce herd immunity to reduce the number of adult hepatitis A incidences in a short time. Thus, additional studies must be conducted to evaluate an optimal vaccination strategy to allow maximum effectiveness given the situation in South Korea [33-36]. It is also necessary to explore the number of hepatitis A incidences in young adults by consistently monitoring patient occurrence patterns, risk factors, and the cost of hepatitis A in the country.

When interpreting the results of the present study, the estimated cost of hepatitis A was conservative in many respects. As the cost and number of hepatitis incidences were estimated by extracting NHI claims data that had disease codes associated with hepatitis A, undiagnosed cases of non-specific hepatitis A and hepatitis A reported as generic hepatitis to avoid cumbersome reporting procedures were excluded from the estimated results of the disease cost in this study. With respect to the cost of productive losses, the number of non-productive days (i.e., absent days) other than inpatient days and outpatient visit days could not be estimated because of a lack of relevant data. For direct and indirect costs, the cost of reduced quality of life because of hepatitis A and psychological costs could not be estimated because of the difficulty of converting the two aforementioned costs into a monetary value. Finally, the present study failed to consider the cost of public health measures in case of a hepatitis A outbreak such as epidemiological investigations. Such limitations suggest that the results of the present study may have underestimated the actual costs of hepatitis A infections. Furthermore, even if fatalities caused by hepatitis A and the transplantation probability of a fulminant hepatitis patient changed by 50% of the base value, the total societal cost of the disease changed by 20%, indicating that the two aforementioned factors have limited influences on the study results.

Furthermore, there were limitations in the data to construct the cost and incidence rate for each clinical pathway by gender. Thus, the study result could not be rendered with respect to gender. Even in the case of indirect costs, we did not differentiate the data by gender and calculated the indirect cost based on the average data of both genders. Considering the fact that the number of hepatitis A incidences in males was approximately 50% higher than that of females [37], we predict the total indirect costs estimated through the present study to be a slightly conservative estimated.

Another limitation of the study is that we did not use a consistent method to calculate the cost and number of hepatitis A incidences when estimating the total societal cost of hepatitis A. We also used many assumptions in our calculations. However, such limitations are difficult to avoid in burden of disease studies given the lack of data that show the overall economic burden of the disease. In addition, because factors that may potentially have a strong influence on the study result were analyzed through sensitivity tests, we believe that we have addressed our shortcomings accordingly. The fact that the number of fatalities caused by hepatitis A and the number of liver transplantations estimated in the study were estimated through its research design is another cautionary note regarding the interpretation of the study results.

In the future, if we accounted for other hepatitis A costs not captured in the present study, we would have a more accurate estimate of the real costs of hepatitis A. The total societal costs of hepatitis A estimated in the study were lower than those of chronic diseases with high prevalence, including general cardiovascular diseases and cancer. However, the indirect cost of hepatitis A because of productive loss from premature death was shown to be relatively high. Thus, reducing indirect rather than direct costs would be more significant when implementing an intervention strategy for hepatitis A. In addition, the study’s clear presentation of the benefits of preventing a single case of hepatitis A will be useful in aiding communication among experts, public health authorities, and citizens.

## Figures and Tables

**Table 1.** Estimated number of hepatitis A infections by clinical pathways and age groups in South Korea, 2008

Age (yr)	Clinical pathways of hepatitis A infections								Total	Incidence (per 100,000)
	I	II	III	IV	V	VI	VII	VIII		
0-4	55 (21.32)	114 (44.19)	88 (34.03)	0 (0.09)	1 (0.22)	0 (0.02)	0 (0.11)	0 (0.03)	258 (0.85)	11.4
5-9	145 (39.94)	108 (29.75)	108 (29.87)	0 (0.09)	1 (0.19)	0 (0.02)	0 (0.10)	0 (0.03)	363 (1.20)	12.8
10-14	270 (30.79)	197 (22.46)	405 (46.17)	1 (0.07)	3 (0.29)	0 (0.03)	1 (0.15)	0 (0.04)	877 (2.90)	26.1
15-19	410 (25.18)	387 (23.77)	820 (50.38)	2 (0.10)	5 (0.32)	1 (0.03)	3 (0.16)	1 (0.05)	1,628 (5.38)	49.7
20-29	1,425 (13.39)	1,891 (17.77)	7,238 (68.01)	8 (0.07)	46 (0.43)	5 (0.04)	23 (0.22)	7 (0.06)	10,643 (35.20)	148.2
30-39	1,486 (13.63)	2,230 (20.46)	7,095 (65.08)	12 (0.11)	45 (0.41)	5 (0.04)	23 (0.21)	7 (0.06)	10,902 (36.05)	131.6
40-49	641 (18.86)	997 (29.34)	1,732 (50.95)	9 (0.28)	11 (0.33)	1 (0.03)	6 (0.16)	2 (0.05)	3,399 (11.24)	40.7
50-59	422 (34.34)	481 (39.14)	313 (25.47)	9 (0.77)	2 (0.17)	0 (0.02)	1 (0.08)	0 (0.03)	1,229 (4.06)	20.4
60-69	194 (32.50)	254 (42.55)	139 (23.23)	9 (1.45)	1 (0.16)	0 (0.02)	0 (0.08)	0 (0.02)	597 (1.97)	15.6
70-79	87 (31.64)	84 (30.55)	95 (34.63)	8 (2.77)	1 (0.24)	0 (0.02)	0 (0.12)	0 (0.04)	275 (0.91)	11.6
80+	13 (19.12)	11 (16.18)	41 (60.25)	3 (3.75)	0 (0.41)	0 (0.04)	0 (0.20)	0 (0.06)	68 (0.22)	8.2
Total	5,149 (17.03)	6,755 (22.34)	18,075 (59.77)	60 (0.20)	115 (0.38)	12 (0.04)	58 (0.19)	17 (0.06)	30,240 (100.00)	62.2

Values are presented as number (%).

**Table 2.** Estimated total costs per capita of hepatitis A infections by clinical pathways and age groups in South Korea, 2008

Age (yr)	Clinical pathways of hepatitis A infections								Weighted average
	I	II	III	IV	V	VI	VII	VIII	
0-4	96	197	1,390	161,044	8,006	168,904	88,734	199,871	934
5-9	102	185	1,664	212,308	8,006	220,168	88,734	251,135	1,002
10-14	125	207	1,788	268,582	8,006	276,442	88,734	307,409	1,460
15-19	131	216	2,072	319,681	8,587	328,075	89,977	359,068	1,897
20-29	156	224	2,489	366,602	8,822	375,221	90,592	406,197	2,677
30-39	191	259	2,759	376,504	9,170	385,455	91,501	416,406	2,921
40-49	174	295	3,055	324,248	9,343	333,363	91,952	364,303	3,046
50-59	158	346	2,806	219,468	9,266	228,510	91,751	259,455	2,785
60-69	145	362	2,912	90,487	9,097	99,368	91,311	130,324	2,323
70-79	131	329	1,995	2,391	8,006	10,250	88,734	41,218	1,041
80+	165	187	2,192	2,391	8,006	10,250	88,734	41,218	1,714
Weighted average	162	259	2,610	232,672	8,974	361,136	91,001	392,099	2,674

Unit: 1 US dollar.

**Table 3.** Estimated total direct and indirect costs of hepatitis A infections by clinical pathways in South Korea, 2008

	Clinical pathways of hepatitis A infections								Total (%)
	I	II	III	IV	V	VI	VII	VIII	
Direct costs									
Medical costs									
Costs paid by health insurance	356	600	16,548	86	562	72	2,976	434	21,633 (26.75)
Costs paid by patients									
For covered services	142	486	5,707	14	94	12	712	77	7,245 (8.96)
For non-covered services	37	203	5,659	26	169	22	1,287	185	7,588 (9.38)
Supplementary care costs	0	0	7722	15	85	11	99	16	7,949 (9.83)
Transportation costs	64	161	765	1	6	1	16	0	1,014 (1.25)
Subtotal	599	1,450	36,401	143	917	117	5,089	713	45,428 (56.17)
Indirect costs									
Number of outpatient visit	12,661	15,903	33,636	0	367	47	1,417	0	64,031
Days of hospitalization	0	0	193,044	384	2,132	273	2,472	410	198,715
Costs of productive loss									
Due to morbidity	234	300	10,768	19	118	15	154	21	11,630 (14.38)
Due to mortality	0	0	0	13,740	0	4,030	0	6,045	23,815 (29.45)
Subtotal	234	300	10,768	13,759	118	4,045	154	6,067	35,445 (43.83)
Total (%)	833 (1.03)	1,750 (2.16)	47,169 (58.32)	13,902 (17.19)	1,034 (1.28)	4,162 (5.15)	5,244 (6.48)	6,779 (8.38)	80,873 (100.00)

Unit: 1,000 US dollar.

**Table 4.** Estimated total cost of hepatitis A infections by clinical pathways and age groups in South Korea, 2008

Age (yr)	Clinical pathways of hepatitis A infections								Total	(%)
	I	II	III	IV	V	VI	VII	VIII		
0-4	5	22	122	36	4	9	25	17	241	(0.30)
5-9	15	20	181	71	6	15	31	26	364	(0.45)
10-14	34	41	724	157	21	71	114	119	1,280	(1.58)
15-19	54	83	1,699	519	45	171	235	281	3,089	(3.82)
20-29	222	423	18,016	2,801	406	1,728	2,086	2,806	28,488	(35.23)
30-39	284	578	19,575	4,367	414	1,741	2,066	2,821	31,847	(39.38)
40-49	112	294	5,290	3,070	103	369	509	605	10,351	(12.80)
50-59	67	166	879	2,073	19	47	94	80	3,424	(4.23)
60-69	28	92	404	784	9	9	43	18	1,387	(1.71)
70-79	11	28	190	18	5	1	29	4	286	(0.35)
80+	2	2	90	6	2	0	12	2	117	(0.14)
Total (% of Total)	833 (1.03)	1,750 (2.16)	47,169 (58.32)	13,902 (17.19)	1,034 (1.28)	4,162 (5.15)	5,244 (6.48)	6,779 (8.38)	80,873 (100.00)	(100.00)

Unit: 1,000 US dollar.

**Table 5.** Outcomes of sensitivity analyses on estimated total costs of hepatitis A infections

	Clinical pathways of hepatitis A infections								Total	Difference with base case (%)
	I	II	III	IV	V	VI	VII	VIII		
Baseline value case	833	1,750	47,169	13,902	1,034	4,162	5,244	6,779	80,873	-
Sensitivity analysis 1	833	1,750	47,285	1,873	1,034	4,162	5,244	6,779	68,960	-14.7
Sensitivity analysis 2	833	1,750	47,052	25,931	1,034	4,162	5,244	6,779	92,786	14.7
Sensitivity analysis 3	833	1,750	47,169	13,902	1,340	5,392	2,622	3,390	76,397	-5.5
Sensitivity analysis 4	833	1,750	47,169	13,902	729	2,933	7,865	10,169	85,349	5.5
Sensitivity analysis 5	833	1,750	47,285	1,873	1,340	5,392	2,622	3,390	64,484	-20.3
Sensitivity analysis 6	833	1,750	47,052	25,931	729	2,933	7,865	10,169	97,262	20.3

Unit: 1,000 US dollar.

Sensitivity analysis 1; 50% of base value on fatality of hepatitis A infection.

Sensitivity analysis 2; 150% of base value on fatality of hepatitis A infection.

Sensitivity analysis 3; 50% of base value on transplantation probability in fulminant hepatitis A infection.

Sensitivity analysis 4; 150% of base value on transplantation probability in fulminant hepatitis A infection.

Sensitivity analysis 5; sensitivity analysis 1 + sensitivity analysis 3.

Sensitivity analysis 6; sensitivity analysis 2 + sensitivity analysis 4.
