# Supplementary material for: The costs of hepatitis A infections in South Korea
Source: Epidemiol Health. 2014 Aug 18;36:e2014011. doi: 10.4178/epih/e2014011 (PMC4154348; doi:10.4178/epih/e2014011)
Supplement: Supplementary file 1 [file epih-36-e2014011-supplemetal data.pdf]

Title: The costs of hepatitis A infections in Republic of Korea

Authors:

Kyohyun Kim[1]

Baek-Geun Jeong[2]

Moran Ki[3]

Mira Park[4]

Jin Kyung Park[5]

Bo Youl Choi[6]

Weon-Seob Yoo[7]

1. Research institute, Health Insurance Review & Assessment Service, Seoul, Korea.
2. Department of Preventive Medicine, School of Medicine, Gyeongsang National University, Jinju, Korea
3. Department of Cancer Control and Policy, Graduated School of Cancer Science and Policy, National Cancer Center, Goyang, Korea
4. Department of Preventive Medicine, School of Medicine, Eulji University, Daejeon, Korea
5. International Vaccine Institute, Seoul, South Korea
6. Department of Preventive Medicine, College of Medicine, Hanyang University, Seoul, South Korea
7. Regional Cardiocerebrovascular Center, Chungnam National University Hospital. Daejeon, South Korea

* Corresponding author:

Weon-Seob Yoo

Tel: +82-42-280-8946

Fax: +82-42-280-8946

E-mail address: wwssuu@gmail.com

Key words

- Cost of illness
- Hepatitis A
- Republic of Korea

This work was supported by grants from Korea Centers for Disease Control and Prevention.

Abstract

**Objectives:**

Recently, the incidence of hepatitis A infection among young adults was increasing in Republic of Korea. Although the universal vaccination has been often suggested to mitigate the problem, its rationale was not well understood. Estimating the societal costs of hepatitis A infections might support the development of intervention strategies.

**Methods:**

We classified the hepatitis A infection into 8 clinical pathways and estimated the number of occurrences and costs per case for each clinical pathway by using the National Health Insurance claims data, several national survey data, and assumptions based on previous studies.

To catch the total costs of hepatitis A infection, both direct and indirect costs were estimated. We included the medical costs, supplement care costs, and traffic costs as the direct costs and productive loss costs due to treatment or premature death as the indirect costs. The indirect costs were estimated with the human capital approach. All costs are adjusted to 2008 year.

**Results:**

30,240 cases were identified as hepatitis A infections in 2008, and the total costs of hepatitis A infections were 80,873 million won (2.7 million won per case). The direct and indirect costs were

56.2% and 43.8% of total cost, respectively. Those aged 20-39 accounted for 71.3% of the total cases and 74.6% of the total costs. The medical costs per capita were lowest in 0-4 age group and highest in 20-29 age group.

**Conclusions:**

The true costs might be higher than our results regarding the non-captured costs and intangible costs of hepatitis A infections costs. This study could provide the evidence for developments of cost-effective interventions to control the hepatitis A infections.

Key words: Cost of illness, Hepatitis A infection, Republic of Korea

**연구배경**

A형 간염은 황달, 열, 식욕부진 등을 증상으로 하는 급성 감염성 질환으로 주로 사람간 전파와 오염된 음식 또는 물을 통해 전파된다 [1]. 전세계적으로 매년 1.4백만 건의 A형 간염이 발생하며, 이로 인한 경제적 부담은 15억~30억 달러로 추계되고 있다 [2].

1990년대 이후, 세계의 많은 지역에서 사회경제적 수준 및 위생 수준의 향상으로 어린 시절의 자연감염 기회가 감소하면서, 인구 전반의 항체 양성률이 낮아지고 성인인구 중 감수성자가 증가하는 A형 간염의 역학적 변천이 보고되고 있다 [3-5]. 소아와는 달리 성인의 A형 간염은 대개 유증상 감염이고 치명적인 결과를 초래할 수 있기 때문에, 성인인구 중 감수성자 증가는 전세계적인 공중보건 이슈 중 하나이다 [6].

최근 10년 동안 한국도 A형 간염의 역학적 변천으로 인하여 성인인구 중 상당수가 A형 간염항체가 없는 것으로 보고되어 이에 대한 우려의 목소리가 커지고 있다 [7-10]. 한국 질병관리본부의 표본감시자료에 따르면 2008년 A형 간염 발생건수는 2004년에 비하여 20배 정도 증가한 약 8000건 이었고, 이중 약 80%가 20-39세 성인인구에서 발생하여, A형 간염 관리대책 보완이 시급히 필요한 상황임을 보여주고 있다 [11].

한국의 A형 간염 관리를 위한 방법으로 예방접종이 제시되고 있으나, A형 간염으로 인한 질병부담, 예방접종전략의 비용-효과성 등 예방접종사업을 위한 정책적 근거는 부족한 상황이다 [12,13].

이 연구는 2008년 한국 A형 간염의 사회경제적 비용을 제시하여 A형 간염에 대한 보건당국과 사회적 관심을 유도하고 비용-효과적인 A형 간염 관리대책을 마련하는데 필요한 근거를 제공하기

위해 수행되었다.

**연구방법**

1. 연구설계 및 연구자료

이 연구는 발생 건에 기초한 접근법(incidence based approach)을 사용하여 2008년 한국에서 발생한 A형 간염의 질병비용부담을 추계하였다. 발생 건에 기초한 접근법은 질병 1건에 대하여 발생 시점부터 완치 또는 사망 시점까지의 질병비용을 산출하는 방법으로, 1건의 질병을 예방하였을 때 발생하는 편익을 예측하는데 유용한 정보를 제공한다 [14,15]. A형 간염의 질병비용부담은 다음과 같은 세 단계를 거쳐 추계하였다.

① A형 간염의 임상경로를 8가지로 구분한 후, 연령 군별로 각 임상경로의 발생확률을 계산하였다. 이 확률에 연령 군별 A형 간염 발생건수를 곱하여 연령 군별, 임상경로별 A형 간염의 발생건수를 산출하였다.

② A형 간염 1 건에 대한 질병비용부담을 직접비용과 간접비용으로 구분하여 연령 군별, 임상경로별로 산출하였다.

③ 연령별, 임상경로별 발생건수(①의 산출물)와 1인당 질병비용부담(②의 산출물)을 곱하여 A형 간염으로 인한 전체 질병비용부담을 산출하였다.

- 국민건강보험 진료비 청구자료 설명

2008년 1개년 동안 한국에서 발생한 A형 간염 발생건수와 비용을 추계하기 위해 국민건강보험 진료비 청구자료를 사용하였다. 국민건강보험공단은 국민건강보험 가입자의 인구학적 정보, 상병명, 진료일수, 진료비, 사망여부 등의 정보가 포함된 자료를 전산관리하고 있다. 이 자료를 활용하면 특정 질병을 대상으로 국가차원의 질병부담 연구가 가능하며, 이미 천식, 손상, 뇌졸중, B형 간염에 대한 질병부담연구가 보고되었다 [16-19].

이 연구는 국민건강보험 청구자료를 활용하여 2008년 한 해 동안 주상병명 또는 부상병명이 A형 관련 상병코드(ICD $10^{th}$ version, B15, B15.0, B15.9)로 청구된 8만여 건의 청구자료 전수를 추출하여 30,240 건의 개인단위 자료로(person based data)로 변환하였다. 이러한 개인단위 자료는 환자 1명에 대한 각기 다른 청구 건을 종합하여, 개인단위로 전체 치료기간 및 치료비용을 측정가능하게 한다. A형 간염은 1회 감염만으로 면역력이 생기는 질환으로 이렇게 변환된 개인 중심자료는 A형 간염 1건에 대한 자료와 같다고 볼 수 있다.

분석을 위한 자료는 건강보험심사평가원에 요청하여 개인식별자가 암호화 처리된 자료로 분석자료를 제공받았으며, 이 연구수행과 관련된 사항은 을지대학병원 기관윤리심의위원회의 승인(IRB No. 09-09)을 받았다.

2. A형 간염의 연령별, 임상경로별 발생확률 및 발생건수

- 이 연구는 선행연구를 바탕으로 하여 A형 간염의 임상경로를 입원 여부, 전격성 간염 발생 여

부, 간이식 여부, 사망 여부에 따라 상호 배타적인 8가지로 구분하였다(Figure 1). 임상경로 I 과 II 는 외래 치료만으로 완치된 A형 간염이며 각각 의원과 병원을 방문한 것이다. 임상경로 III 과 IV 는 비전격성 간염으로 입원 후 각각 생존 또는 사망한 경우이다. 임상경로 V 와 VI 는 전격성 간염으로 입원하였으나 여러 사정으로 간이식 수술을 받지 못하고 각각 생존 또는 사망한 경우이다. 임상경로 VII 과 VIII 는 전격성 간염으로 입원 후 수술을 받고 각각 생존 또는 사망한 경우이다.

각 임상경로의 발생확률은 <figure 1>의 각 임상경로가 나누어지는 각 지점의 확률을 곱하여 계산 할 수 있다. 예를 들어, A형 간염의 입원확률, 입원환자의 전격성 간염 발생확률, 전격성 간염 발생환자의 간이식수술 시행률, 이식 받은 환자의 생존율을 알 수 있다면, 각 단계의 확률을 곱하여 A형 간염 환자에서 "임상경로 8"이 발생할 확률을 계산할 수 있다. 국민건강보험 청구자료와 선행연구결과를 활용하여 각 분기점의 연령 군별 확률을 산출하였으며, 연령 군별 확률을 산출할 수 없는 경우에는 각 분기점의 확률이 모든 연령에서 같다고 가정하였다.

- A형 간염의 연령 군별 외래 방문확률(의원, 병원)과 입원확률은 국민건강보험 진료비 청구자료를 활용하여 산출하였다. 국내 자료가 없는 입원환자의 전격성 간염 발생확률은 Bauch 등이 보고한 1.1% 를 적용하였다 [20]. 전격성 간염 환자의 간이식수술 시행률(37.1%), 간이식수술 환자의 치명률(23.1%), 간이식수술을 받지 않은 환자의 치명률(9.1%)은 국내 연구결과를 이용하였다 [21]. 전격성 간염이 아닌 A형 간염 환자가 사망할 확률은 A형 간염 환자 사망률과 A형 간염환자 중 전격성 간염으로 사망할 확률의 차를 산출하였다 [21,22].

- 이렇게 계산한 확률에 청구자료의 연령 군별 A형 간염 발생건수를 곱하여 연령 군별, 임상경로별 A형 간염의 발생건수를 산출하였다. 또, 연령군별 A형 간염발생률 산출을 위한 기준인구는 통계청 2008년 추계인구를 사용하였다.

3. A형 간염의 비용 추정

– A형 간염의 질병비용은 연령군별, 임상경로별로 직접비와 간접비를 산출하였다. 직접비는 의료비, 간병비, 교통비를 포함하며, 간접비는 치료기간 동안의 생산성 손실비용과 조기사망으로 인한 생산성 손실 비용을 포함한다. 모든 비용은 2008년을 기준으로 산출하였고, 2008년 6월 매매기준율(1 USD ≒ 1000 KRW)을 적용하여 미국달러(USD)로 표시하였다.

- 직접비

의료비는 보험자 부담비용(costs paid by insurance), 환자 본인 일부 부담비용(costs paid by patient as cost-sharing), 환자 비급여 부담비용(costs paid by patient for not covered services)으로 구분하였다. 보험자 부담비용과 환자 본인 일부 부담비용은 국민건강보험 급여대상 서비스에 대한 비용으로(costs for covered services) 청구자료와 선행연구를 이용하여 산출하였다. A형 간염의 환자 비급여 부담비용은 "2007년도 건강보험환자의 본인부담 진료비 실태조사" 결과를 이용하여 "보장되는 서비스에 대한 부담비용" 대비 "보장되지 않는 서비스에 대한 부담비용" 비를 적용하여 산출하였다 [23]. 또한, 연구에서 사용한 국민건강보험 청구자료는 외래투약 비용을 포함하지 않

아, 2007년 국민건강보험 통계연보 자료 중 외래 투약일수와 외래 내원일수의 비를 이용하여 계산한 내원일당 외래 투약일수에 외래 투약일당 투약비용을 곱하여 총 외래투약 비용을 간접적으로 산출하였다 [24].

임상경로 Ⅰ과 Ⅱ의 의료비용은 청구자료 중 입원진료를 받은 적이 없는 A형 간염 발생 건을 선택하여 연령 군별로 산출하였다. 임상경로 Ⅲ과 IV의 의료비용은 청구자료 중 입원진료를 받은 적이 있고, A형 간염으로 인한 진료비가 상위 1.1%에 속하지 않으며, 치료결과가 “생존” 또는 “사망”인 건을 선택하여 산출하였다. 이는 A형 간염 입원환자 중 전격성 간염 분율이 1.1%라는 선행 연구결과와 전격성 간염은 진료비가 높을 것이라는 가정을 근거한 것이다 [20].

임상경로 Ⅴ와 VI의 의료비용은 한국의 선행연구를 바탕으로 산출한 입내원일수와 임상경로 Ⅲ의 입내원일당 의료비용을 곱하여 산출하였다 [21]. 이렇게 산출한 임상경로 Ⅴ와 VI의 의료비용은 각각 7,157 USD, 9,169 USD 이었다.

임상경로 Ⅶ의 의료비용은 간이식 비용과 관련한 한국의 선행연구를 바탕으로 산출하였다. Yang 등이 보고한 B형 간염 환자의 간이식 비용, Ha의 논문에서 산출한 B형 간염 환자의 간이식 비용 대비 기타 환자의 간이식 비용의 비, 수가인상률을 반영하여 A형 간염 환자의 간이식 비용을 2008년 기준으로 산출하였다 [19,25]. 이렇게 산출한 임상경로 Ⅶ의 간이식 첫 해 입원 비용, 간이식 첫 해 외래 비용, 간이식 둘째 해 외래 비용은 각각 72,850 USD, 4,203 USD, 9,279 USD이었고, 전체 비용은 86,332 USD 이었다.

임상경로 Ⅷ의 의료비용은 간이식 환자의 입원일수와 임상경로 Ⅶ의 의료비용을 바탕으로 산출하였다. 간이식 수술 후 생존한 A형 간염환자의 입원일수와 임상경로 Ⅶ의 입원 비용을 이용하여

산출한 입원일당 비용에 간이식 수술 후 사망한 A형 간염환자의 입원일수를 곱하여 산출하였다 [21]. 산출된 임상경로 Ⅷ의 의료비용은 40,246 USD 이었다.

A형 간염환자의 비의료비는 의료기관 방문 시 발생하는 교통비와 입원환자 간호를 위한 간병비를 포함하였다. 교통비는 외래 방문 일수와 외래 방문 일당 교통비의 곱과 입원 시 교통비의 합으로 계산하였다. 방문 1일당 교통비는 국가단위의 조사자료인 "2005년 국민건강영양조사자료"를 이용하여 의원외래, 병원외래, 입원 건에 대하여 왕복비용을 산출한 뒤, 소비자 물가지수를 반영하여 2008년 기준으로 계산하였다. 의원외래와 병원외래의 방문 1일당 교통비는 각각 5.0 USD, 10.1 USD 이었고, 입원 1건당 교통비는 23.5 USD 이었다. 외래 방문일수의 산출방법은 아래의 간접비 산출방법에서 설명하였다.

간병비는 의료비 외에 추가적인 간병에 대한 직접비용으로 국내 사정을 반영하여 입원기간 동안 매일 정액으로 발생한다고 가정하였다 [26,27]. 2008년 일당 간병비는 시장가격을 반영하여 40 USD로 가정하였다.

- 간접비

간접비는 인적자본 접근법을 사용하여 생산성 손실비용을 추계하였다. 치료기간 동안의 생산성 손실비용은 A형 간염으로 인한 비생산 일수와 일 평균 임금을 곱하여 산출하였다. 비생산 일수는 입원 일수와 외래 방문 일수의 1/3을 합하여 산출하였다 [17,18,26].

임상경로 Ⅰ-Ⅳ의 외래방문 일수 및 입원일수는 국민건강보험 청구자료로 산출하였다. 임상경로

Ⅴ와 VI의 입원일수는 선행연구에 근거하여 각각 18.5일, 23.7일로 하였고, 외래 방문 일수는 이 입원일수에 임상경로 Ⅲ의 입원일당 외래 방문 일수 비를 곱하여 각각 3.2일, 4.1일로 하였다[21] . 임상경로 Ⅶ과 Ⅷ의 입원일수는 같은 선행연구에 근거하여 각각 42.9일, 23.7일로 하였다. 임상경로 Ⅶ의 외래방문은 간이식 후 첫 6개월 동안에는 월 2회, 이 후 12개월 동안은 월 1회, 그 이후에는 외래방문이 없다고 가정하여, 24.6일을 외래방문일수로 하였다. 임상경로 Ⅷ는 외래방문이 없다고 가정하였다. 2008년 기준 연령별 일 평균 임금은 2006년 연령별 월 급여 총액을 일 평균 임금으로 재구성한 뒤, 소비자물가지수 상승률과 연령별 고용률을 곱하여 산출하였다 [28]. 생산활동에 참여하는 연령층은 15세-69세로 가정하였다. 임금통계자료가 생성되지 않는 15세의 일 평균 임금은 16세의 일 평균 임금으로 대체하였고, 61-69세 연령의 일 평균 임금은 60세의 일 평균 임금으로 대체하였다. 14세 이하 또는 70세 이상 환자의 외래 방문 시 손실되는 비용은 이 연령층의 보호자를 40세 여성으로 가정하여 이 연령의 일 평균 임금과 고용률을 적용하여 추계하였다.

조기사망에 의한 생산성 손실비용은 A형 간염으로 사망한 환자가 사망하지 않았을 경우 69세까지 벌어들일 것으로 예상되는 미래 기대소득을 2008년 기준의 연령별 연평균 임금, 연령별 고용률, 자연사망률을 고려하여 연간 할인율 5%를 적용하여 2008년 화폐가치로 추계하였다 [28]. 2008년 기준 연령별 연평균 임금은 통계청의 2006년 임금에 연도별 소비자물가지수 상승률을 곱하여 계산하였고, 자연사망률은 통계청 자료를 이용하였다. 예를 들어, 2008년 사망한 30세 성인의 생산성 손실비용은 378,000 USD로 추계되었다.

(Equation 1.)

4. 민감도 분석

민감도 분석을 위해 비전격성 A형 간염의 사망률과 전격성 A형 간염의 간이식 수술시행률을 고려하였다. 각 수치가 기본값에서 50% 또는 150% 변할 때, A형 간염의 질병비용부담 크기의 변화를 결과에 제시하였다.

결과.

- A형 간염 발생건수

건강보험 청구자료를 통해 확인한 2008년 한국의 A형 간염 발생건수는 총 30,240 건이었고, 발생률은 10만 명당 62.2건으로 나타났다 (Table 1). 20대 및 30대 연령의 발생건수는 각각 10,643건, 10,902 건으로 전체 발생건수의 71.3%를 차지하였으며, 발생률은 각각 10만 명당 148.2건, 131.6건으로 가장 높은 발생률을 보였다. 임상경로별 발생건수는 임상경로 Ⅲ(전격성 간염 없이 입원치료로 완치된 경우)가 18,075건으로 전체 발생 건수의 59.8%를 차지하여 가장 흔한 임상경로였다. 전격성 간염이 발생한 A형 간염은 202건으로 추정되었으며, 이 가운데 75 건이 간이식을 받은 것으로 추정되었다.

각 연령군의 임상경로별 발생확률은, 입원을 필요로 하는 임상경로 Ⅲ-Ⅷ가 발생할 확률의 경우 20대 연령에서 68.8%로 가장 높았고, 5-9세 및 60대 연령에서 각각 30.3%와 25.0%로 가장 낮았다. 사망 건에 해당하는 임상경로 IV, VI, Ⅷ가 발생할 확률은 0-4세에서 0.1%로 가장 낮았으며, 80

세 이상에서 3.9%로 가장 높았다.

- A형 간염 1건당 비용

직접비용과 간접비용을 합한 A형 간염 1건당 총비용은 2,674 USD로, 연령군별 비용은 0-4세 연령군에서 934 USD 로 가장 낮았으며, 40-49세 연령군에서 3,046 USD으로 가장 높았다 (Table 2). 임상경로별 1건당 총비용은 외래 진료만으로 완치되는 임상경로 Ⅰ과 Ⅱ의 경우 각각 162 USD, 259 USD 으로 가장 낮았고, 간이식으로 완치되는 임상경로 Ⅶ의 경우 91,001 USD이었으며, 사망에 이르는 임상경로 IV, VI, Ⅷ에서 20만 USD로 가장 높았다. 가장 많은 환자가 발생한 20대의 임상경로 Ⅲ에 해당하는 A형 간염의 경우 1 건당 총비용이 2,489 USD으로 추정되었다.

A형 간염 1건당 총비용 2,674 USD 가운데 직접비용은 1,502 USD이었고, 직접비용 중 의료비용는 1,206 USD이었다. 같은 임상경로의 A형 간염이라도 연령에 따라 발생하는 1건당 의료비용은 다른 것으로 나타났다. 임상경로 Ⅲ의 경우 1건당 발생하는 의료비용이 20-39세에서 0-4세에서 발생하는 비용보다 약 1.5배 높았다.

- A형 간염의 전체 비용

2008년 한 해 동안 A형 간염으로 발생한 전체 비용은 총 80,873천 USD로 추정되었다 (Table 3). A형 간염으로 인한 전체 비용 가운데 직접비용과 간접비용은 각각 56.2%와 43.8%를 차지하였다. 직접비용의 80.3%가 의료비용 이였으며, 나머지 19.3%가 간병비용과 교통비용이었다. 의료비용의

59.3%는 보험자 부담비용이었고, 40.7%는 환자 부담비용이었다. 간접비용의 1/3은 의료이용으로 인한 생산성 손실 비용이었고, 나머지 2/3은 조기사망으로 인한 생산성 손실비용이었다.

임상경로 Ⅲ은 A형 간염으로 인한 전체 비용의 58.3%를 차지하여 8가지 임상경로 가운데 가장 많은 비용을 차지하였다. A형 간염으로 인한 총 외래방문 횟수는 64,031회, 총 입원일수는 198,715 일 (544년) 이었다.

2008년 발생한 A형 간염으로 인한 전체 비용의 연령별 분포는 <Table 4>와 같다. 전체 비용 가운데 30대 연령군에서 발생한 비용이 39.4%로 가장 많았으며, 20대부터 40대까지의 연령군에서 발생한 비용은 전체 비용의 87.4%를 차지하였다.

- 민감도 분석결과

민감도 분석결과는 <Table 5>와 같다. 비전격성 A형 간염의 사망률과 전격성 간염의 간이식 수술 시행률이 모두 50% 증가하였을 때(Sensitivity analysis 6), A형 간염의 전체 비용은 97,262천 USD 로 기초분석결과 보다 20.3% 높은 값을 나타냈다. 비전격성 A형 간염의 사망률과 전격성 간염의 간이식 수술 시행률이 모두 50% 감소하였을 때(Sensitivity analysis 5) A형 간염의 전체 비용은 64,484천 USD로 기초분석결과 보다 20.3% 낮은 값을 나타냈다. 비전격성 A형 간염 사망률의 변화가 간이식 시행률 변화보다 A형 간염의 전체 비용에 더 큰 영향을 미쳤다.

고찰

이 연구는 건강보험 청구자료를 바탕으로 발생 건에 기초한 접근법을 사용하여, 2008년 한국에서 발생한 A형 간염의 질병비용부담을 추계하였다. 연구결과 2008년 A형 간염 발생 건수는 30,240 건이었고, 사회경제적 측면에서의 질병비용은 80,873천 USD 이었으며, 직접비용 중 의료비용과 비의료비용은 각각 45.1%, 11.1% 였고, 간접비용은 43.8%로 나타났다. 20-39세 연령이 전체 발생 건수의 71.3%, 사회경제적 비용의 74.6%를 차지하였다. A형 간염 1 건당 질병비용 2,674 USD이었다. A형 간염의 총 질병비용부담은 한국에서 보고된 다른 질환(암, 뇌졸중, 천식, 손상)에 비하여 적은 것으로 나타났지만 [17,18,27,29], 다른 소화기 질환과 비교하였을 때, A형 간염의 질병비용은 소화기 감염병 중 하나인 로타바이러스에 의한 질병비용 보다는 높고, 과민성 대장증후군에 의한 질병비용 보다는 낮은 것으로 나타났다 [34-35]. 2005년 간접비를 포함한 로타바이러스로 인한 질병비용은 약 13.3 백만 USD 로 보고되었으며, 물가상승률을 반영한 2008년 질병비용은 14.6 백만 USD 로, 이 값과 비교하였을 때 A형 간염의 질병비용은 약 5.5배 더 높은 것으로 나타났다. 과민성 대장증후군에 의한 2008년 질병비용은 585.4 백만 USD 로, 이 값은 A형 간염 질병비용의 7.2배에 해당하였다. 로타바이러스 및 과민성 대장증후군에 의한 질병비용 가운데 간접비가 차지하는 분율은 각각 5%, 25%로, A형 간염에서 간접비의 분율(44%)이 이들 질환보다 큰 것으로 나타났다.

이 연구를 통해 확인된 2008년 A형 간염 발생 건수 30,240건은 국민건강보험공단 진료비 청구자료의 주상병명 또는 부상병명만으로 A형 간염을 확인된 것으로, 의료기관의 상병명 입력 행태에 따라 발생 건수 및 이에 따른 질병비용이 영향을 받을 수 있다. 별도의 연구로 진행된 작업에

따르면 2002년부터 2008년까지 11개의 종합병원급 의료기관에서 의무기록으로 확인된 A형 간염 발생 건수(3,081 건)와 건강보험자료에서 확인된 발생 건수(2,928 건)에 큰 차이가 없는 것으로 보고되어, 국민건강보험공단 진료비 청구자료에 근거한 A형 간염 발생 건수를 의료기관을 이용한 A형 간염 발생건수로 활용하는데 큰 무리가 없다고 판단하였다 [37]. 그러나 종합병원 외 의료기관의 A형 간염 상병명 입력과 관련된 행태는 확인하지 못하였기 때문에, 보다 정확한 A형 간염 발생 건수 추정은 향후 추가적인 연구가 필요하다.

연구결과 2008년 한국 A형 간염의 질병비용 규모는 GDP 대비 0.008%, OECD 국민의료비 대비 0.12%로 추정되었다. 2000년 출판된 미국의 연구는 A형 간염의 총 질병비용을 488.8백만 USD 로 보고하였는데, 이 수치는 해당연도 GDP 대비 0.006%, OECD 국민의료비 대비 0.044%에 해당하며, 한국의 A형 간염 질병비용보다 상대적으로 작았다 [36]. 이는 이 연구의 연구대상 연도의 A형 간염 발생률이 미국보다 약 2배 높았다는 것으로 일부 설명된다. 한편 한국의 A형 간염 비용 가운데 간접비가 차지하는 분율은 44%, 미국을 대상으로 한 연구결과는 74%로, 한국의 A형 간염 비용이 상대적으로 보수적으로 추계되었음을 알 수 있었다. 이 연구의 A형 간염 질병비용을 직접적으로 비교할 만한 미국 이외 다른 국가들에 관한 자료는 확인하지 못하였다.

20-39세 연령군에서 발생하는 A형 간염이 전체 간염건수와 질병비용부담의 70% 이상을 차지하고 있기 때문에 질병비용부담을 낮추기 위해서는 젊은 성인에서 발생하는 A형 간염에 대한 관리가 필요한 것으로 보인다. A형 간염 예방에 효과가 입증된 예방접종을 젊은 성인에 우선적으로 적용하는 방안을 생각할 수 있지만, 소아 대상의 국가예방접종 도입만으로도 무증상감염자에 의한 전파 차단과 집단면역효과로 단기간에 성인의 A형 간염 발생건수를 감소시켰다고 보고하고

있어, 한국의 상황에서 효과적인 예방접종 전략이 무엇인지는 추가적인 연구가 필요하다 [30-33]. 한국에서 발생하는 A형 간염의 위험요인에 대한 확인 및 환자발생 양상과 질병비용의 지속적인 모니터링을 통하여 젊은 성인에서 A형 간염 발생을 낮출 수 있는 방안을 모색할 필요가 있다.

이 연구의 결과를 해석할 때에는 추계된 A형 간염의 질병비용부담이 몇 가지 측면에서 적게 추계되었다는 점을 주의해야 한다. 건강보험 데이터베이스에서 진단명이 A형 간염 관련 코드로 되어 있는 자료만을 추출하여 A형 간염의 발생 건수와 발생비용을 추계하였으므로, 비 특이적인 증상으로 A형 간염으로 진단받지 못한 경우와 신고절차에 따른 불편함으로 A형 간염의 진단명을 기타 간염으로 하였을 경우는 질병비용부담 추계 결과에서 누락되게 된다. 또, 생산성 손실비용은 입원 일수 또는 외래 방문 일수 외에 발생할 수 있는 비 생산 일수는(예, 결근 일수) 관련 자료의 부족으로 추계하지 못하였다. 직접비와 간접비 외의 A형 간염 인한 삶의 질 악화, 유쾌하지 않은 기분 등에 의한 비용은 화폐가치로 환산하는데 어려움이 있어 추계하지 못하였다. 마지막으로 A형 간염 유행이 발생한 경우 시행되는 역학조사 등의 공중보건조치에 대한 비용도 이 연구에서 고려하지 못하였다. 이러한 제한점들은 A형 간염으로 인한 실제 질병비용부담보다 이 연구의 결과가 저추계되었을 가능성이 있음을 시사하며, 이런 저추계 요인들이 있는 가운데, A형 간염으로 인한 사망률과 전격성 간염 환자의 간이식 시행확률이 기본 값에서 50% 변하더라도 전체 질병비용부담의 값은 20% 내에서 변하여 이들 요인이 연구결과에 미치는 영향력은 제한적임을 확인하였다.

한편 임상경로별 발생 확률 및 해당 비용을 성별로 구축하는데 자료의 한계가 있어, 연구결과를 성별로 산출하지 못하였다. 간접비의 경우에도 성별로 자료를 구축하지 않고 남녀의 평균 자

료를 근거로 간접비를 산출하였는데, 여성보다 남성의 A형 간염 발생 건이 약 50% 높다는 점을 고려하면 [38], 이 연구를 통해 추정된 간접비용은 다소 저추계되었을 것이라고 추정된다.

또한, A형 간염의 전체 질병비용부담 추계 시, 비용과 발생건수를 산출하는 방법이 일관적이지 못하였고, 여러 가정이 사용되었다는 점도 연구의 제한점이다. 그러나 이러한 제한점은 질병비용부담을 전체적으로 보여주는 자료가 부족한 현실에서 질병부담연구나 경제성평가 연구에서 피하기 힘든 제한점이고, 연구결과에 큰 영향을 줄 수 있는 요인은 민감도 분석에서 살펴보았기 때문에 어느 정도 보완이 되었으리라 생각된다. 연구에서 추정된 A형 간염으로 인한 사망 건수 및 간이식 건수가 연구설계에 의해 추정된 건수라는 것도 연구결과의 해석 시 주의해야 하는 사항이다.

향후 이 연구에서 적용한 비용추계방법에 추가로 확인되는 A형 간염 비용을 반영한다면 좀더 정확한 A형 간염의 질병비용부담을 추계할 수 있을 것이다. 이 연구에서 추계된 A형 간염의 질병비용부담은 일반적인 심혈관질환, 암질환 등 유병률이 높은 만성질환에 비하여 낮았으나, 조기사망으로 생산성 손실로 인한 간접비용은 상대적으로 높은 것으로 나타나 예방관리전략 도입 시 직접적인 비용보다는 간접적인 비용을 감소시키는 데 의미가 있을 것으로 예상된다." 아울러 A형 간염 1건을 예방하였을 때 발생되는 편익을 명확하게 제시한 연구결과는 전문가, 보건당국, 국민들 사이의 의사소통에 도움을 줄 것으로 기대한다.

References


1. Purcell RH, Emerson SU, eds. *Ch. 8 natural history and experimental models*. 3rd ed. Viral hepatitis, ed. Thomas Hc, Lemon S, Zuckerman Aj. 2005, Wiley-Blackwell: Massachusetts. 109-125.
2. World Health Organization. Hepatitis a. *Department of Communicable Disease Surveillance and Response document* 2000:
3. Jacobsen KH, Koopman JS. Declining hepatitis a seroprevalence: A global review and analysis. *Epidemiology and Infection* 2004; 132(6): 1005-1022.
4. Jacobsen KH, Koopman JS. The effects of socioeconomic development on worldwide hepatitis a virus seroprevalence patterns. *International Journal of Epidemiology* 2005; 34(3): 600-609. English
5. Catton MG, Locarnini SA, eds. *Ch. 7 epidemiology*. 3rd ed. Viral hepatitis, ed. Thomas Hc, Lemon S, Zuckerman Aj. 2005, Wiley-Blackwell: Massachusetts. 92-108.
6. Hendrickx G, Van Herck K, Vorsters A, Wiersma S, Shapiro C, Andrus JK, et al. Has the time come to control hepatitis a globally? Matching prevention to the changing epidemiology. *Journal of Viral Hepatitis* 2008; 15: 1-15. English
7. Song YB, Lee JH, Choi MS, Koh KC, Paik SW, Yoo BC, et al. The age-specific seroprevalence of hepatitis a virus antibody in korea. *Korean J Hepatol.* 2007; 13(1): 27-33.
8. Kang JH, Lee KY, Kim CH, Sim D. Changing hepatitis a epidemiology and the need for vaccination in korea. *Asian Pacific Journal of Allergy and Immunology* 2004; 22(4): 237-242.
9. Kang CI, Choi CM, Park TS, Lee DJ, Oh MD, Choe KW. Incidence and seroprevalence of hepatitis a virus infections among young korean soldiers. *Journal of Korean Medical Science* 2007; 22(3): 546-548. English
10. Sohn YM, Rho HO, Park MS, Park JH, Choi BY, Ki M, et al. The changing epidemiology of hepatitis a in children and the consideration of active immunization in korea. *Yonsei Medical Journal* 2000; 41(1): 34-39. English
11. Korea Center for Disease Control and Prevention. *Viral hepatitis statistics*. Communicable disease sentinel surveillance [cited 2009 9.20]; Communicable disease information system]. Available from: http://stat.cdc.go.kr/.
12. Lee D, Cho YA, Park Y, Hwang JH, Kim JW, Kim NY, et al. Hepatitis a in korea: Epidemiological shift and call for vaccine strategy. *Intervirology* 2008; 51(2): 70-74.
13. Kim JH. Recent epidemiological status and vaccination of hepatitis a in korea. *Journal of the Korean Medical Association* 2008; 51(2): 110-118.
14. Tarricone R. Cost-of-illness analysis what room in health economics? *Health Policy* 2006; 77(1): 51-63.

15. Rice D. Cost-of-illness studies: Fact or fiction? *The Lancet* 1994; 344(8936): 1519-1520.
16. Park C, Kang H, Kwon I, Kang D, Jung H. Cost-of-illness study of asthma in korea: Estimated from the korea national health insurance claims database. *J Prev Med Public Health* 2006; 39(5): 397-403.
17. Park K, Lee J, Kim Y, Kim Y, Kim J. The socioeconomic cost of injuries in south korea. *J Prev Med Public Health* 2009; 42(1): 5-11.
18. Lim S, Kim H, Nam C, Chang H, Jang Y, Kim S, et al. Socioeconomic costs of stroke in korea: Estimated from the korea national health insurance claims database. *J Prev Med Public Health* 2009; 42(4): 251-260.
19. Yang BM, Kim CH, Kim JY. Cost of chronic hepatitis b infection in south korea. *Journal of Clinical Gastroenterology* 2004; 38(10): S153-S157. English
20. Bauch CT, Anonychuk AM, Pham BZ, Gilca V, Duval B, Krahn MD. Cost-utility of universal hepatitis a vaccination in canada. *Vaccine* 2007; 25(51): 8536-8548.
21. Kim JM, Lee YS, Lee JH, Kim W, Lim KS. Clinical outcomes and predictive factors of spontaneous survival in patients with fulminant hepatitis a.
. *Korean Journal of Hepatology* 2008; 14(4): 474-482.
22. Jacobs RJ, Greenberg DP, Koff RS, Saab S, Meyerhoff AS. Regional variation in the cost effectiveness of childhood hepatitis a immunization. *Pediatric Infectious Disease Journal* 2003; 22(10): 904-914.
23. Kim J, Lee H, Jeong H, *Survey on out-of-pocket payment of national health insurance, 2007* 2008, National Health Insurance Corporation.
24. National Health Insurance Corporation, *2007 national health insurance statistical yearbook*. 2008, National Health Insurance Corporation: Seoul.
25. Ha HS. National health insurance coverage and household burden of liver transplatation (author's translation) *The Korean Journal of Hepatology* 2005; 11(2): 111-114.
26. Jung Y, Ko S. The socioeconomic cost of diseases in korea. *J Prev Med Public Health* 2006; 39(6): 499-504.
27. Kim J, Hahm M, Park E, Park J, Kim S, Kim S. Economic burden of cancer in south korea for the year 2005. *J Prev Med Public Health* 2009; 42(3): 190-198.
28. Statistics Korea. *Employment, labor and wage*. Korean Statistical Information Service [cited 2009 9.1]; Available from: http://www.kosis.kr/.
29. Park C, Kang H, Kwon I, Kang D, Jung H. Cost-of-illness study of asthma in korea: Estimated from the korea national health insurance claims database *J Prev Med Public Health* 2006; 39(5): 397-403.
30. Dagan R, Leventhal A, Anis E, Slater P, Ashur Y, Shouval D. Incidence of hepatitis a in israel following universal immunization of toddlers. *Jama-Journal of the American Medical Association* 2005; 294(2): 202-210. English
31. Wasley A, Samandari T, Bell BP. Incidence of hepatitis a in the united states in the era of

vaccination. *Jama-Journal of the American Medical Association* 2005; 294(2): 194-201. English
32. Vacchino MN. Incidence of hepatitis a in argentina after vaccination. *Journal of Viral Hepatitis* 2008; 15: 47-50. English
33. Dominguez A, Oviedo M, Carmona G, Jansa JM, Borras E, Salleras L, et al. Epidemiology of hepatitis a before and after the introduction of a universal vaccination programme in catalonia, spain. *Journal of Viral Hepatitis* 2008; 15(s2): 51-56.

34. Yang BM, et al. The societal cost of rotavirus infection in South Korea. Korean Journal of Pediatrics, 2008; 51(9): 977-986.
35. Choi MG, et al. Calculation of the burden of irritable bowel syndrome, National evidence based healthcare collaborating agency, 2011, (Korean, unpublished report) (URL:http://www.neca.re.kr/center/researcher/report_view.jsp?boardNo=GA&seq=3&q=626f6172644e6f3d4741, access date : 2014.6.22.)
36. BERGE, Jeremy J., et al. The cost of hepatitis A infections in American adolescents and adults in 1997. *Hepatology*, 2000, 31.2: 469-473.
37. Ki. MR, et al. Economic Analysis of Vaccination and Development of Guidelines for Control of Hepatitis A Virus Infection, and Epidemiologic Analysis and Development of Control Strategy of Hepatitis C Virus Infection. Center for disease control and prevention, 2009 (Korean, unpublished report)
38. Korea center for disease control and prevention. Disease web statistics system. (http://is.cdc.go.kr/nstat/index.jsp)

**Table 1. Estimated numbers of hepatitis A infections by its clinical pathways and age groups in South Korea, 2008**

| Age groups | | Clinical pathways of hepatitis A infections | | | | | | | | Total | (% of Total) | Incidence (per 100000) |
|---|---|---|---|---|---|---|---|---|---|---|---|---|
| | | Ⅰ | Ⅱ | Ⅲ | Ⅳ | Ⅴ | Ⅵ | Ⅶ | Ⅷ | | | |
| 0-4 | Cases | 55 | 114 | 88 | 0 | 1 | 0 | 0 | 0 | 258 | (0.85) | 11.4 |
| | (%) | (21.32) | (44.19) | (34.03) | (0.09) | (0.22) | (0.02) | (0.11) | (0.03) | (100.00) | | |
| 5-9 | Cases | 145 | 108 | 108 | 0 | 1 | 0 | 0 | 0 | 363 | (1.20) | 12.8 |
| | (%) | (39.94) | (29.75) | (29.87) | (0.09) | (0.19) | (0.02) | (0.10) | (0.03) | (100.00) | | |
| 10-14 | Cases | 270 | 197 | 405 | 1 | 3 | 0 | 1 | 0 | 877 | (2.90) | 26.1 |
| | (%) | (30.79) | (22.46) | (46.17) | (0.07) | (0.29) | (0.03) | (0.15) | (0.04) | (100.00) | | |
| 15-19 | Cases | 410 | 387 | 820 | 2 | 5 | 1 | 3 | 1 | 1,628 | (5.38) | 49.7 |
| | (%) | (25.18) | (23.77) | (50.38) | (0.10) | (0.32) | (0.03) | (0.16) | (0.05) | (100.00) | | |
| 20-29 | Cases | 1,425 | 1,891 | 7,238 | 8 | 46 | 5 | 23 | 7 | 10,643 | (35.20) | 148.2 |
| | (%) | (13.39) | (17.77) | (68.01) | (0.07) | (0.43) | (0.04) | (0.22) | (0.06) | (100.00) | | |
| 30-39 | Cases | 1,486 | 2,230 | 7,095 | 12 | 45 | 5 | 23 | 7 | 10,902 | (36.05) | 131.6 |
| | (%) | (13.63) | (20.46) | (65.08) | (0.11) | (0.41) | (0.04) | (0.21) | (0.06) | (100.00) | | |
| 40-49 | Cases | 641 | 997 | 1,732 | 9 | 11 | 1 | 6 | 2 | 3,399 | (11.24) | 40.7 |
| | (%) | (18.86) | (29.34) | (50.95) | (0.28) | (0.33) | (0.03) | (0.16) | (0.05) | (100.00) | | |
| 50-59 | Cases | 422 | 481 | 313 | 9 | 2 | 0 | 1 | 0 | 1,229 | (4.06) | 20.4 |
| | (%) | (34.34) | (39.14) | (25.47) | (0.77) | (0.17) | (0.02) | (0.08) | (0.03) | (100.00) | | |
| 60-69 | Cases | 194 | 254 | 139 | 9 | 1 | 0 | 0 | 0 | 597 | (1.97) | 15.6 |
| | (%) | (32.50) | (42.55) | (23.23) | (1.45) | (0.16) | (0.02) | (0.08) | (0.02) | (100.00) | | |
| 70-79 | Cases | 87 | 84 | 95 | 8 | 1 | 0 | 0 | 0 | 275 | (0.91) | 11.6 |
| | (%) | (31.64) | (30.55) | (34.63) | (2.77) | (0.24) | (0.02) | (0.12) | (0.04) | (100.00) | | |
| 80+ | Cases | 13 | 11 | 41 | 3 | 0 | 0 | 0 | 0 | 68 | (0.22) | 8.2 |
| | (%) | (19.12) | (16.18) | (60.25) | (3.75) | (0.41) | (0.04) | (0.20) | (0.06) | (100.00) | | |
| Total | Cases | 5,149 | 6,755 | 18,075 | 60 | 115 | 12 | 58 | 17 | 30,240 | (100.00) | 62.2 |
| | (%) | (17.03) | (22.34) | (59.77) | (0.20) | (0.38) | (0.04) | (0.19) | (0.06) | (100.00) | | |

**Table 2. Estimated total costs per capita of hepatitis A infections by its clinical pathways and age groups in South Korea, 2008 (Unit: 1 USD)**

| Age groups | Clinical pathways of hepatitis A infections | | | | | | | | Weighted Average |
|---|---|---|---|---|---|---|---|---|---|
| | Ⅰ | Ⅱ | Ⅲ | Ⅳ | Ⅴ | Ⅵ | Ⅶ | Ⅷ | |
| 0-4 | 96 | 197 | 1,390 | 161,044 | 8,006 | 168,904 | 88,734 | 199,871 | 934 |
| 5-9 | 102 | 185 | 1,664 | 212,308 | 8,006 | 220,168 | 88,734 | 251,135 | 1,002 |
| 10-14 | 125 | 207 | 1,788 | 268,582 | 8,006 | 276,442 | 88,734 | 307,409 | 1,460 |
| 15-19 | 131 | 216 | 2,072 | 319,681 | 8,587 | 328,075 | 89,977 | 359,068 | 1,897 |
| 20-29 | 156 | 224 | 2,489 | 366,602 | 8,822 | 375,221 | 90,592 | 406,197 | 2,677 |
| 30-39 | 191 | 259 | 2,759 | 376,504 | 9,170 | 385,455 | 91,501 | 416,406 | 2,921 |
| 40-49 | 174 | 295 | 3,055 | 324,248 | 9,343 | 333,363 | 91,952 | 364,303 | 3,046 |
| 50-59 | 158 | 346 | 2,806 | 219,468 | 9,266 | 228,510 | 91,751 | 259,455 | 2,785 |
| 60-69 | 145 | 362 | 2,912 | 90,487 | 9,097 | 99,368 | 91,311 | 130,324 | 2,323 |
| 70-79 | 131 | 329 | 1,995 | 2,391 | 8,006 | 10,250 | 88,734 | 41,218 | 1,041 |
| 80+ | 165 | 187 | 2,192 | 2,391 | 8,006 | 10,250 | 88,734 | 41,218 | 1,714 |
| Weighted Average | 162 | 259 | 2,610 | 232,672 | 8,974 | 361,136 | 91,001 | 392,099 | 2,674 |

**Table 3. Estimated total direct and indirect costs of hepatitis A infections by its clinical pathways in South Korea, 2008 (Unit: 1,000 USD)**

| | Clinical pathways of hepatitis A infections | | | | | | | | Total | (% of Total ) |
|---|---|---|---|---|---|---|---|---|---|---|
| | Ⅰ | Ⅱ | Ⅲ | Ⅳ | Ⅴ | Ⅵ | Ⅶ | Ⅷ | | |
| **Direct costs** | | | | | | | | | | |
| Medical costs | | | | | | | | | | |
| Costs paid by health insurance | 356 | 600 | 16,548 | 86 | 562 | 72 | 2,976 | 434 | 21,633 | (26.75) |
| Costs paid by patients | | | | | | | | | | |
| For covered services | 142 | 486 | 5,707 | 14 | 94 | 12 | 712 | 77 | 7,245 | (8.96) |
| For non-covered services | 37 | 203 | 5,659 | 26 | 169 | 22 | 1,287 | 185 | 7,588 | (9.38) |
| Supplementary care costs | 0 | 0 | 7,722 | 15 | 85 | 11 | 99 | 16 | 7,949 | (9.83) |
| Transportation costs | 64 | 161 | 765 | 1 | 6 | 1 | 16 | 0 | 1,014 | (1.25) |
| Subtotal | 599 | 1,450 | 36,401 | 143 | 917 | 117 | 5,089 | 713 | 45,428 | (56.17) |
| **Indirect costs** | | | | | | | | | | |
| Number of outpatient visit | 12,661 | 15,903 | 33,636 | 0 | 367 | 47 | 1,417 | 0 | 64,031 | - |
| Days of hospitalization | 0 | 0 | 193,044 | 384 | 2,132 | 273 | 2,472 | 410 | 198,715 | - |
| Costs of productive loss | | | | | | | | | | |
| due to morbidity | 234 | 300 | 10,768 | 19 | 118 | 15 | 154 | 21 | 11,630 | (14.38) |
| due to mortality | 0 | 0 | 0 | 13,740 | 0 | 4,030 | 0 | 6,045 | 23,815 | (29.45) |
| Subtotal | 234 | 300 | 10,768 | 13,759 | 118 | 4,045 | 154 | 6,067 | 35,445 | (43.83) |
| Total | 833 | 1,750 | 47,169 | 13,902 | 1,034 | 4,162 | 5,244 | 6,779 | 80,873 | (100.00) |
| (% of Total) | (1.03) | (2.16) | (58.32) | (17.19) | (1.28) | (5.15) | (6.48) | (8.38) | (100.00) | |

**Table 4. Estimated total cost of hepatitis A infections by its clinical pathways and age groups in South Korea, 2008 (Unit: 1,000 USD)**

| Age groups | Clinical pathways of hepatitis A infections | | | | | | | | Total | (% of Total) |
|---|---|---|---|---|---|---|---|---|---|---|
| | Ⅰ | Ⅱ | Ⅲ | Ⅳ | Ⅴ | Ⅵ | Ⅶ | Ⅷ | | |
| 0-4 | 5 | 22 | 122 | 36 | 4 | 9 | 25 | 17 | 241 | (0.30) |
| 5-9 | 15 | 20 | 181 | 71 | 6 | 15 | 31 | 26 | 364 | (0.45) |
| 10-14 | 34 | 41 | 724 | 157 | 21 | 71 | 114 | 119 | 1,280 | (1.58) |
| 15-19 | 54 | 83 | 1,699 | 519 | 45 | 171 | 235 | 281 | 3,089 | (3.82) |
| 20-29 | 222 | 423 | 18,016 | 2,801 | 406 | 1,728 | 2,086 | 2,806 | 28,488 | (35.23) |
| 30-39 | 284 | 578 | 19,575 | 4,367 | 414 | 1,741 | 2,066 | 2,821 | 31,847 | (39.38) |
| 40-49 | 112 | 294 | 5,290 | 3,070 | 103 | 369 | 509 | 605 | 10,351 | (12.80) |
| 50-59 | 67 | 166 | 879 | 2,073 | 19 | 47 | 94 | 80 | 3,424 | (4.23) |
| 60-69 | 28 | 92 | 404 | 784 | 9 | 9 | 43 | 18 | 1,387 | (1.71) |
| 70-79 | 11 | 28 | 190 | 18 | 5 | 1 | 29 | 4 | 286 | (0.35) |
| 80+ | 2 | 2 | 90 | 6 | 2 | 0 | 12 | 2 | 117 | (0.14) |
| Total | 833 | 1,750 | 47,169 | 13,902 | 1,034 | 4,162 | 5,244 | 6,779 | 80,873 | (100.00) |
| (% of Total) | (1.03) | (2.16) | (58.32) | (17.19) | (1.28) | (5.15) | (6.48) | (8.38) | (100.00) | |

**Table 5. Outcomes of sensitivity analyses on estimated total costs of hepatitis A infections (Unit: 1,000 USD)**

| | Clinical pathways of hepatitis A infections | | | | | | | | Total | Difference with base case |
|---|---|---|---|---|---|---|---|---|---|---|
| | Ⅰ | Ⅱ | Ⅲ | Ⅳ | Ⅴ | Ⅵ | Ⅶ | Ⅷ | | |
| Base value case | 833 | 1,750 | 47,169 | 13,902 | 1,034 | 4,162 | 5,244 | 6,779 | 80,873 | - |
| Sensitivity analysis 1 | 833 | 1,750 | 47,285 | 1,873 | 1,034 | 4,162 | 5,244 | 6,779 | 68,960 | -14.7% |
| Sensitivity analysis 2 | 833 | 1,750 | 47,052 | 25,931 | 1,034 | 4,162 | 5,244 | 6,779 | 92,786 | 14.7% |
| Sensitivity analysis 3 | 833 | 1,750 | 47,169 | 13,902 | 1,340 | 5,392 | 2,622 | 3,390 | 76,397 | -5.5% |
| Sensitivity analysis 4 | 833 | 1,750 | 47,169 | 13,902 | 729 | 2,933 | 7,865 | 10,169 | 85,349 | 5.5% |
| Sensitivity analysis 5 | 833 | 1,750 | 47,285 | 1,873 | 1,340 | 5,392 | 2,622 | 3,390 | 64,484 | -20.3% |
| Sensitivity analysis 6 | 833 | 1,750 | 47,052 | 25,931 | 729 | 2,933 | 7,865 | 10,169 | 97,262 | 20.3% |

Sensitivity analysis 1 : 50% of base value on fatality of hepatitis A infection
Sensitivity analysis 2 : 150% of base value on fatality of hepatitis A infection
Sensitivity analysis 3 : 50% of base value on transplantation probability in fulminant hepatitis A infection
Sensitivity analysis 4 : 150% of base value on transplantation probability in fulminant hepatitis A infection
Sensitivity analysis 5 : Sensitivity analysis 1 + Sensitivity analysis 3
Sensitivity analysis 6 : Sensitivity analysis 2 + Sensitivity analysis 4

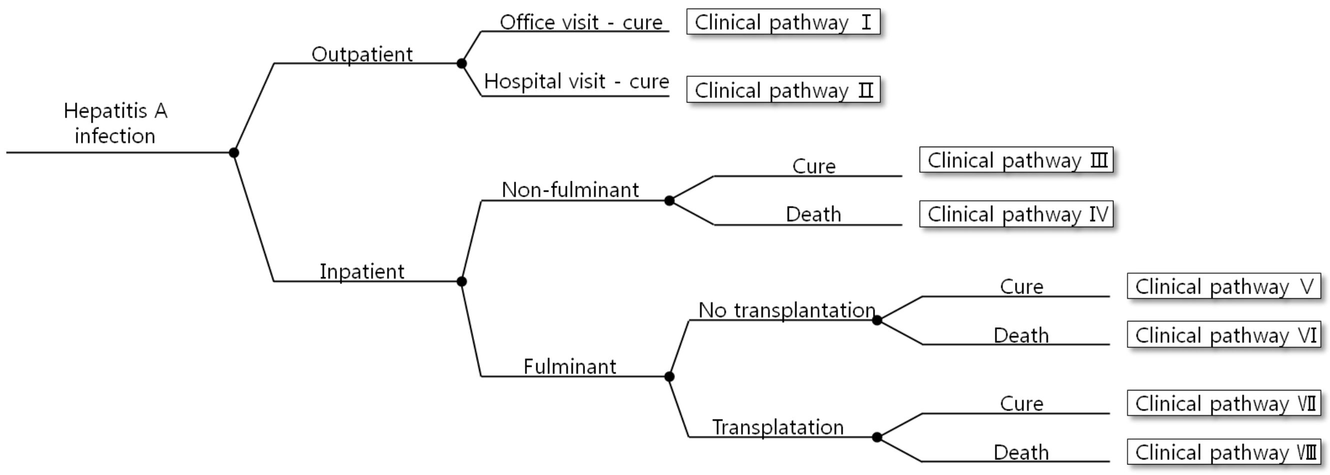


**Figure 1. Eight clinical pathways model of hepatitis A infections**

Equation 1.

$$Costs\ of\ productive\ loss\ due\ to\ mortality = \sum_{i=0} \sum_{\tau=0} \frac{\mathrm{N_i} \times \mathrm{w_{i+\tau}} \times \mathrm{e_{i+\tau}} \times \mathrm{d_{i+\tau}}}{(1+r)^{\tau}}$$

i : Age of morality case

τ : years after death

ω : annual wage rate

e : employment rate

d : natural mortality rate

r : discount rate (0.05)
